# Early diabetic kidney maintains the corticomedullary urea and sodium gradient

**DOI:** 10.14814/phy2.12714

**Published:** 2016-03-20

**Authors:** Haiyun Qi, Thomas S. Nørlinger, Per M. Nielsen, Lotte B. Bertelsen, Emmeli Mikkelsen, Yafang Xu, Hans Stødkilde Jørgensen, Christoffer Laustsen

**Affiliations:** ^1^ Department of Clinical MedicineMR Research CentreAarhus UniversityAarhusDenmark

**Keywords:** Hyperpolarization, kidney, MRI, renal function, type 1 diabetes

## Abstract

Early diabetic nephropathy is largely undetectable before substantial functional changes have occurred. In the present study, we investigated the distribution of electrolytes and urea in the early diabetic kidney in order to explore whether pathophysiological and metabolic changes appear concomitantly with a decreased sodium and urea gradient. By using hyperpolarized ^13^C urea it was possible to measure the essential intrarenal electrolyte gradients and the acute changes following furosemide treatment. No differences in either intrarenal urea or sodium gradients were observed in early diabetes compared to healthy controls. These results indicate that the early metabolic and hypertrophic changes occurring in the diabetic kidney prelude the later functional alterations in diabetic kidney function, thus driving the increased metabolic demand commonly occurring in the diabetic kidney.

## Introduction

The kidneys play a pivotal role in adjusting the balance of water and electrolyte with the corticomedullary sodium, urea, and chloride concentration gradients (Sands and Layton 2009). Even though the kidneys only represent 0.5% of the total body mass, the renal oxygen consumption accounts for 10% of the total oxygen consumption of the human body (Nordquist and Palm 2007). Renal tubular sodium transport is the primary determinant of renal oxygen consumption as the entire plasma is filtered every 30 min, and 99% of the renal filtrate is reabsorbed through O_2_‐dependent active transport (O'Connor 2006; Hansell et al. 2013). The high oxygen consumption manifests as low renal oxygen tension under normal conditions, and thus attempts to increase oxygen delivery via increased renal blood flow is ensued by an increased energetic demand, by increasing the glomerular filtration rate, thus directly leading to kidney hypoxia, a common linkage in the development of kidney diseases (Fine et al. 2000). In diabetic animals, renal hypoxia has been attributed to increased oxygen consumption due to hyperfiltration, resulting in increased tubular sodium reabsorption as well as reduced efficacy of oxygen utilization (Palm et al. 2004; Hansell et al. 2013; Takiyama and Haneda 2014). This appears concurrently with pathogenic tubular growth (structural change) and abnormal hemodynamics resulting in high glomerular perfusion and filtration (Jerums et al. 2010; Vallon 2011; Hansell et al. 2013). A vital challenge in the treatment of diabetic nephropathy is the ability to an early and more reliable identification of high‐risk patients, emphasizing the importance of noninvasive imaging methods for monitoring of renal functional and metabolic alterations in the kidneys. Recent advances in hyperpolarization methodology (Ardenkjaer‐Larsen et al. 2003, 2011) have allowed comprehensive investigations of perfusion, metabolite transport, exchange, and metabolism in vivo with exogenous bioprobes such as [1‐^13^C]pyruvate or [1‐^13^C]urea (von Morze et al. 2012; Laustsen et al. 2013, 2014; Pages et al. 2013), and have recently been introduced for human application (Nelson et al. 2013). Hyperpolarized urea has been shown to be an excellent perfusion marker showing high‐resolution perfusion information in vivo with no background (von Morze et al. 2011, 2012). In the present study, we explore the potential of hyperpolarized [^13^C,^15^N]urea to identify early pathological changes of glomerular hyperfiltration as seen in early diabetic kidney.

## Materials and Methods

Sixteen 10‐week 200 g female Wistar rats were randomly grouped in a diabetic group of six animals and a healthy control group of six animals and a parallel control group of four animals receiving furosemide treatment. Diabetes was induced by an intravenous injection of freshly prepared streptozotocin (STZ; 55 mg/kg body weight, Sigma‐Aldrich, Broendby, Denmark). Rats were considered diabetic, when the blood glucose level exceeded 15 mmol/L at 48 h after injection of STZ. Blood glucose was measured in tail–capillary blood with a ContourXT blood glucose meter (Bayer Diabetes Care, Copenhagen, Denmark). Eight to 10 days after the induction of diabetes, the rats were anesthetized (3% sevoflurane and 2 L/min air) and a tail vein catheter was inserted for injection of 1.5 mL hyperpolarized [^13^C,^15^N]urea polarized in a SPINLab (GE Healthcare, Broendby, Denmark). The MR examination was performed in a 9.4 T preclinical MR system (Agilent, Santa Clara, CA, USA) equipped with a dual tuned ^13^C/^1^H volume rat coil (Doety Scientific, Columbia, SC). Additionally in four healthy rats, the acute changes following 10 mg/kg of furosemide (Furix, Takeda Pharma A/S, Roskilde, Denmark) injection were investigated. Just after the first MR examination was performed, the furosemide was administered intravenously and 20 min after a second MR examination was performed. The temperature was maintained at 35°C, and respiration and peripheral capillary oxygen saturation (SpO_2_) levels were monitored throughout the experiment (Table 1). A ^1^H T_2_‐weighted Fast Spin Echo coronal and axial scan was acquired (TR/TE 3 sec/4 msec, flip 90°/180°, ETL 4, ESP 10 msec, FOV 70‐mm, matrix 128 × 128) as an anatomical ^1^H scout. A 2D ^13^C‐balanced steady‐state free precession imaging sequence was performed every 2 sec, acquiring 40 images in total and was initiated at the start of hyperpolarized [^13^C,^15^N]urea injection with a flip angle = 15°, TR/TE = 4.8 msec/2.4 msec, sw = 20 kHz, FOV = 60 × 60 mm and a 32 × 32 matrix and an axial slice thickness of 10‐mm covering both kidneys. A standard 3D gradient echo sequence was used for thermal ^23^Na MRI, with TR/TE 50 ms/2 ms, sw = 10 kHz, matrix 32 × 32 × 8, FOV 60 mm × 60 mm × 60 mm, with 32 numbers of transients. After the MR examination, a 5–7 mL arterial blood sample, both kidneys and urine were collected for further measurement of laboratory parameters.

**Table 1:** Body weight (BW), kidney weight (KW), blood glucose level (fed state at the day of scan), peripheral capillary oxygen saturation (SpO_2_), and kidney weight per kg rat (KW/BW) in diabetic and control groups (mean of both kidneys)

	Body weight (g)	Kidney weight (g)	Blood glucose level (mmol/L)	SpO_2_ (%)	KW/BW (g/kg)
Control	221.26 ± 2.68	0.78 ± 0.03	6.97 ± 0.39	83.20 ± 2.40	3.52 ± 0.08
Diabetic	219.77 ± 3.93	0.93 ± 0.02^a^	25.07 ± 0.82^a^	86.83 ± 2.24	4.25 ± 0.08^a^

Mean ± SEM of *n* = 5–6/group.

a *P* < 0.05 versus control group.

The mRNA expression of KIM‐1, NGAL, Nqo‐1, IL‐1*β*, TNF‐*α*,* α*‐SMA, and Collagen‐I in inner medulla was quantified with real‐time quantitative PCR (qPCR). Total RNA of cortex was extracted using the Machery–Nagel's NucleoSpin RNA II Kit, and complementary DNA (cDNA) was synthesized with cDNA Synthesis Kit (Thermo Scientific, Hvidovre, Denmark) according to the manufacturer's instructions. qPCR was performed using 100 ng cDNA as a template for PCR amplification, and 18S served as a reference gene. Maxima SYBR Green qPCR Master Mix was used according to the manufacturer's protocols (Stratagene, AH Diagnostics, Aarhus, Denmark). For qPCR experiments, a standard curve was constructed by plotting threshold cycle (Ct values) against serial dilutions of purified PCR product. The sequences of all primers for qPCR were: KIM‐1 (forward, 5′‐CCA CAA GGC CCA CAA CTA TT‐3′ and reverse, 5′‐TGT CAC AGT GCC ATT CCA GT‐3′), NGAL (forward, 5′‐GAT CAG AAC ATT CGT TCC AA‐3′ and reverse, 5′‐TTG CAC ATC GTA GCT CTG TA‐3′), Nqo‐1 (forward, 5′‐GTG GTG ATG GAA AGC AAG GT‐3′ and reverse, 5′‐GCC CGG ATA TTG TAG CTG AA‐3′), IL‐1*β* (forward, 5′‐CAC AGC AGC ATC TCG ACA AGA‐3′ and reverse, 5′‐AAG ACA TAG GTA GCT GCC ACA GC ‐3′), TNF‐*α* (forward, 5′‐GCC CTA AGG ACA CCC CTG AGG GAG C‐3′ and reverse, 5′‐TCC AAA GTA GAC CTG CCC GCA CTC C‐3′), *α*‐SMA (forward, 5′‐CAT CAT GCG TCT GGA CTT GG‐3′ and reverse, 5′‐CCA GGG AAG AAG AGG AAG CA‐3′), Collagen‐I (forward, 5′‐TCA AGA TGG TGG CCG TTA CT‐3′ and reverse, 5′ ‐CAT CTT GAG GTC ACG GCA TG ‐3′), and 18S (forward, 5′‐CAT GGC CGT TCT TAG TTG‐3′, and reverse, 5′‐CAT GCC AGA GTC TCG TTC‐3′). The study complied with the guidelines for use and care of laboratory animals and was approved by the Danish Inspectorate of Animal Experiments.

An amount of 200 *μ*L [^13^C,^15^N]urea (Sigma‐Aldrich), glycerol (Sigma‐Aldrich), AH111501 (GE Healthcare) (6.4 mol/L concentration) mixed ratio (0.30:0.68:0.02) was polarized in a 5T SPINLab (GE Healthcare) to a reproducible polarization of more than 30%. The hyperpolarized urea sample was subsequently rapidly dissolved and transferred to the rats already placed in a 9.4T preclinical MR scanner. Regions‐of‐interest of left and right kidney cortex, medulla, and pelvis were manually segmented to measure the intrarenal distribution normalized to the total renal signal and a region inside the abdominal aorta was used to obtain the arterial input curve (AIF). The ratio between areas under the curve (AUC) for the cortical compartment with the AIF curve was used as a measure of the perfusion of renal perfusion (Johansson et al. 2004; Lau et al. 2015). Normality was assessed with quantile–quantile plots. A value of *P* < 0.05 (*) was considered statistically significant. The statistical analysis was performed in PRISM. Comparisons of animal and kidney weight and blood glucose were analyzed with a two‐tailed Student's *t* test with equal variance, as well as the qPCR data and plasma and urine data. Urea and sodium gradients were analyzed with a two‐way ANOVA, with a Tukey's correction for multiple comparisons. A repeated measure two‐way ANOVA with the same correction was used to compare the effect of furosemide treatment.

## Results

**Figure 1:**
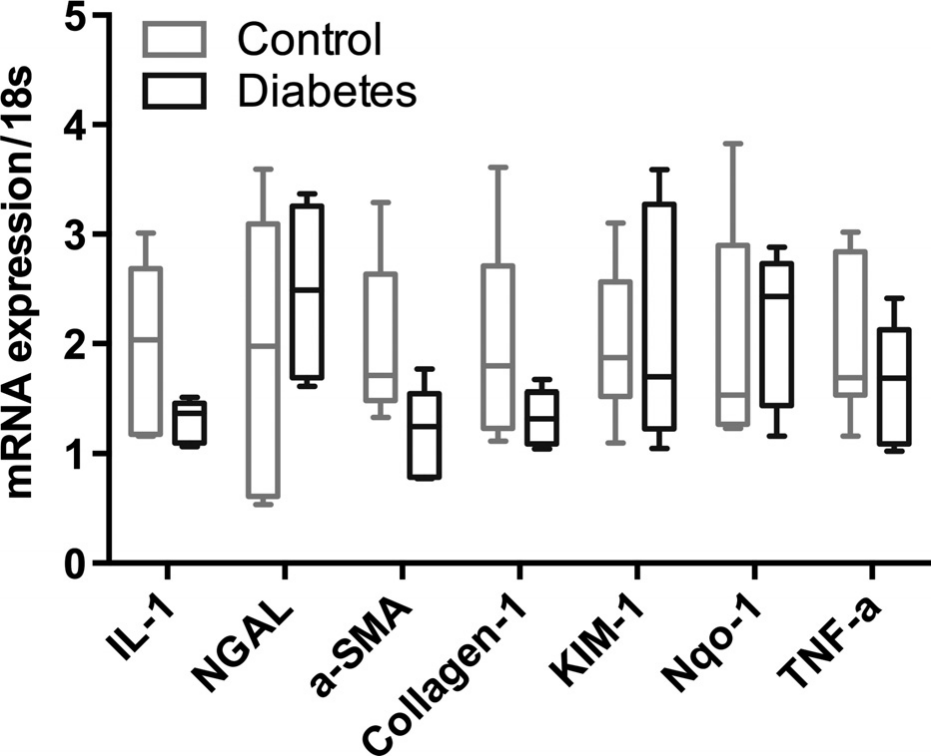
mRNA expression of KIM‐1 is significantly increased in the diabetic group. mRNA expression of NGAL, IL‐1, *α*‐SMA, Nqo‐1, KIM‐1,TNF‐*α*, and collegan‐1 is similar between the control and diabetic groups (median ± min–max).

All six rats receiving STZ injection developed sustained hyperglycemia over the subsequent 48 h. One rat in the control group died on the day of examination and was excluded from the study. Fifteen rats were included in study (diabetic *n* = 6, controls *n* = 5, furosemide *n* = 4). Body weight did not show any difference (*P* = 0.77) between the diabetic group (219.77 ± 3.93 g) and the control group (221.26 ± 2.68 g) at the day of examination. However, kidney weight of the diabetic rats (0.93 ± 0.02 g) was statistically significantly higher compared to the controls (0.78 ± 0.03 g, *P* = 0.0006). The blood glucose level in the diabetic group (25.07 ± 0.82 mmol/L) was persistently higher (*P* < 0.0001) than in controls (6.97 ± 0.39 mmol/L) (Table 1). A statistically significant decreased urinary concentration of urea was found in the diabetic group compared to the control group (*P* = 0.0002), whereas the sodium concentration in the urine was similar between the diabetic group and controls (*P* = 0.23), albeit a tendency was observed toward a urine dilution in the diabetes group. Plasma creatinine was not significantly different between the two groups (*P* = 0.9) (Table 2). The qPCR showed a similar cortical mRNA expression of KIM‐1, NGAL, IL‐1, *α*‐SMA, Nqo‐1, TNF‐*α*, and collegan‐1 between diabetics and controls (Fig. 1).

**Table 2:** Urine urea, urine sodium, and plasma creatinine in diabetic and control groups at the day of the scan

	U‐Urea (mmol/L)	U‐sodium (mmol/L)	P‐creatinine (*μ*mol/L)
Control	626.20 ± 73.83	71.00 ± 20.34	16.80 ± 1.99
Diabetic	218.70 ± 8.21^a^	45.50 ± 6.98	17.20 ± 1.77

Mean ± SEM of *n* = 5–6/group.

a *P* < 0.05 versus control group.

**Figure 2:**
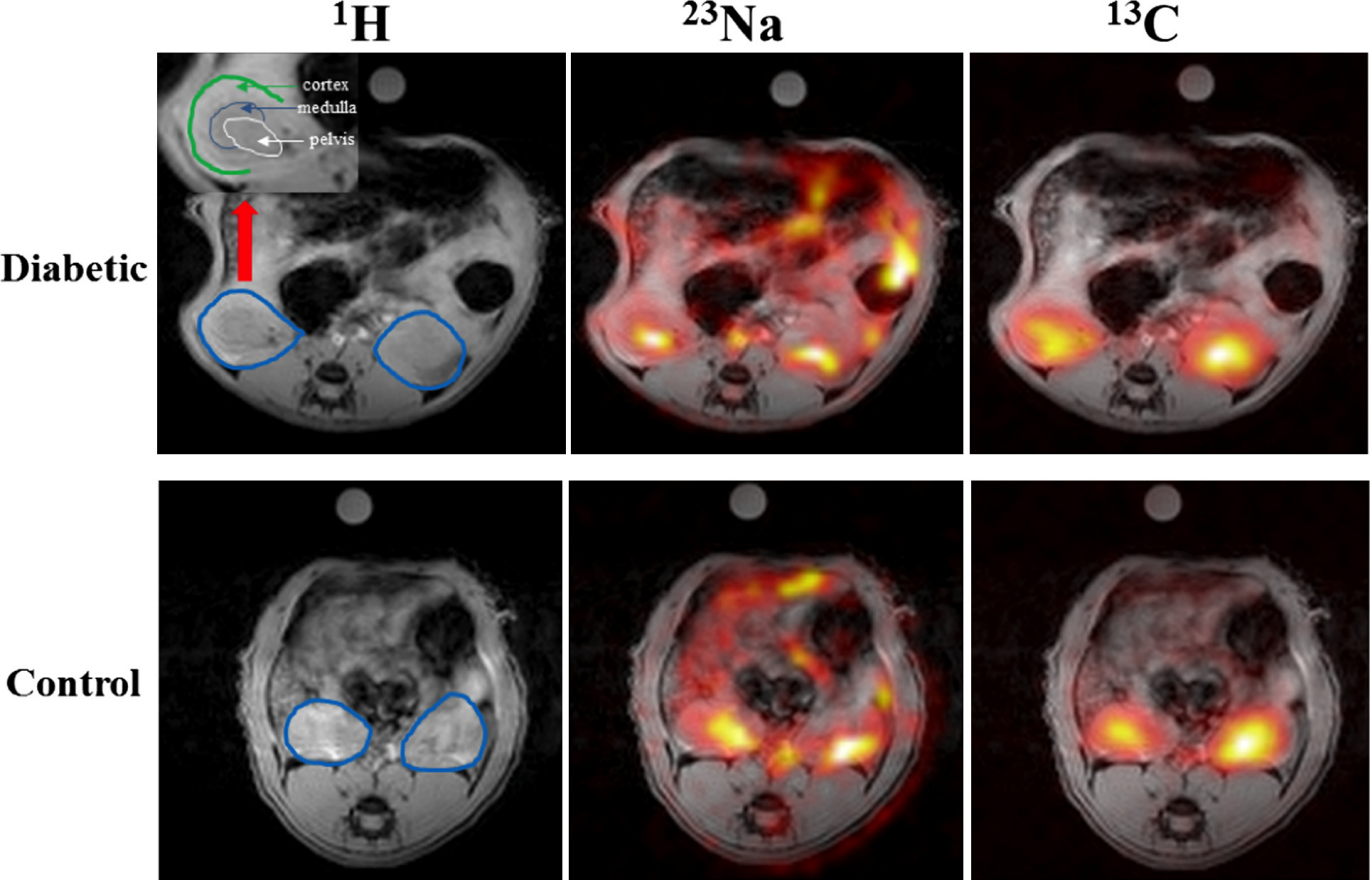
Representation of both kidneys (blue circles) in 1H MR images (left), renal distribution of sodium (^23^Na) (middle) and hyperpolarized ^13^C urea signal (16th image, 32 sec after start of injection) (right) in a diabetic and control animal. The zoomed image in the upper left corner shows the individual renal cortex, medulla, and pelvis in the right kidney (red arrow).

**Figure 3:**
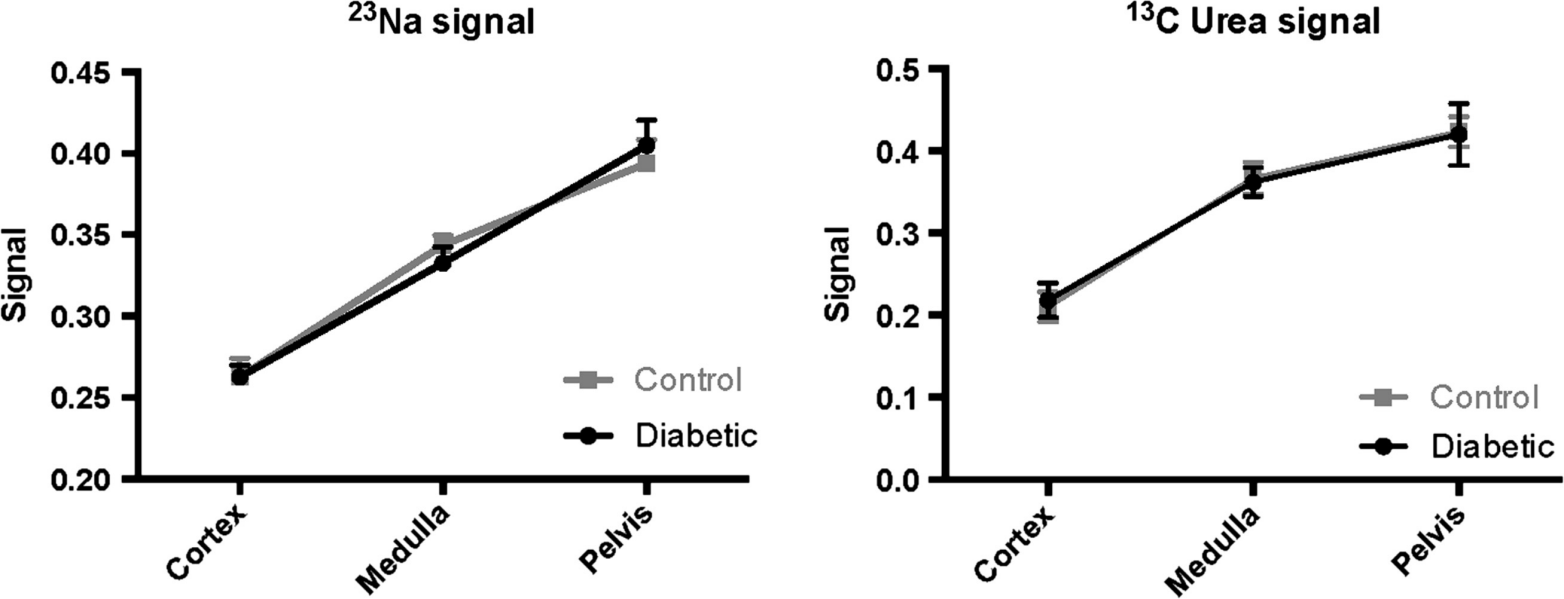
The renal perfusion (renal compartment/aorta signal) shows no significant alterations in the early diabetic kidney compared to control kidneys (mean ± SEM).

**Figure 4:**
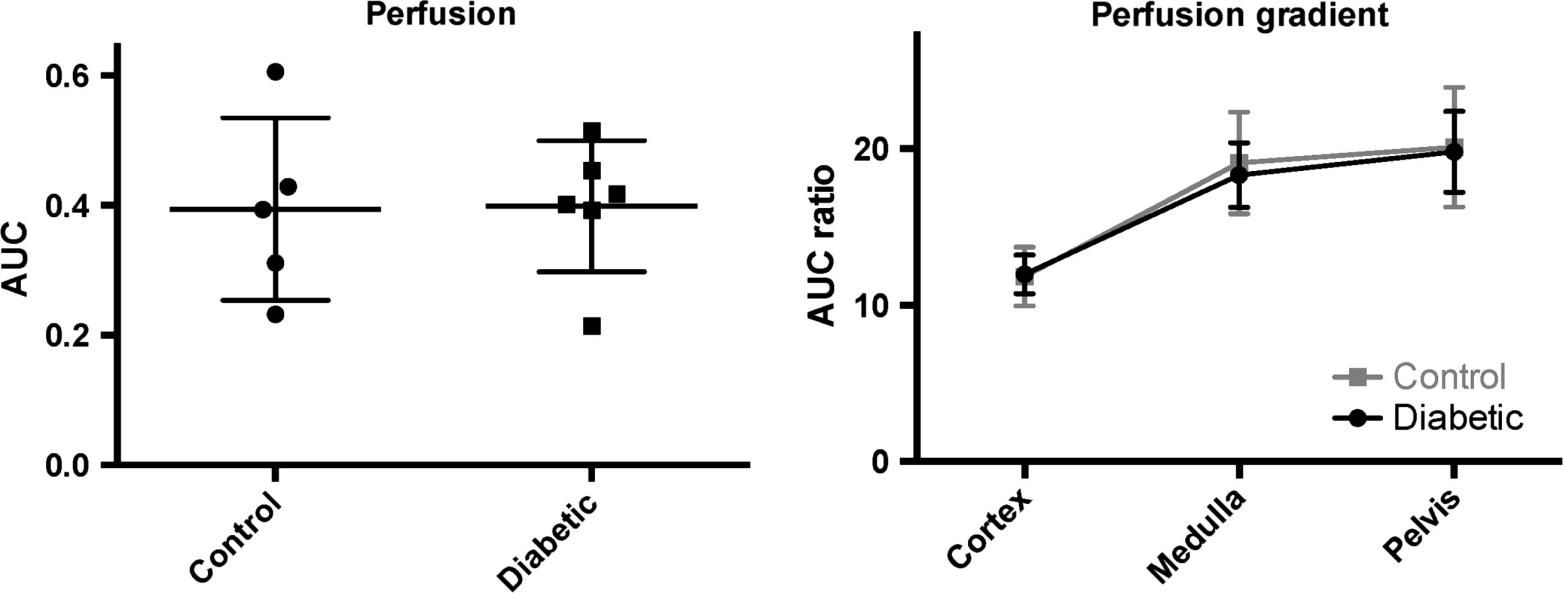
The intrarenal sodium and urea signals (normalized to the total renal signal) show no difference between the diabetic group and the control group, whereas a significant difference is present within each group (mean ± SEM).

**Figure 5:**
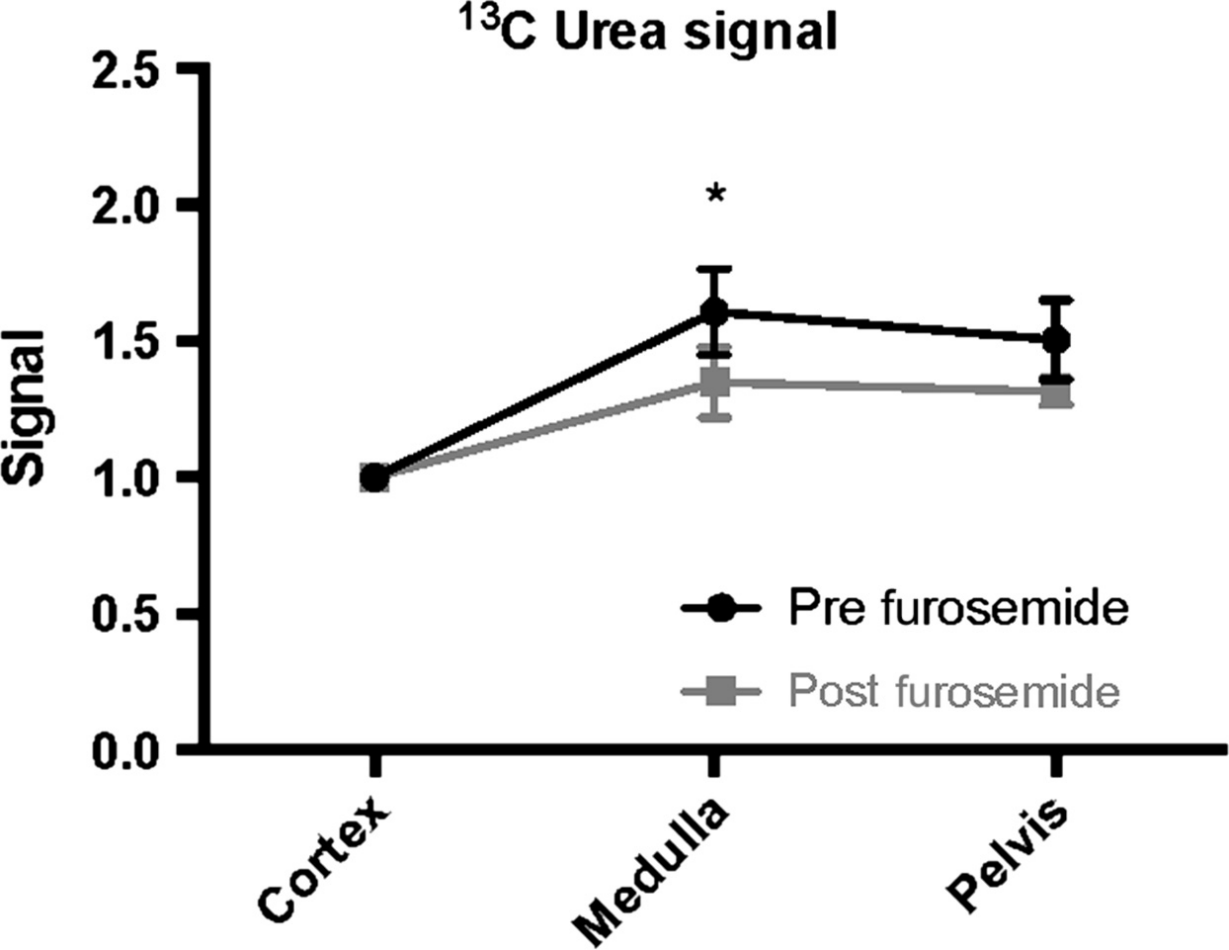
Healthy rats (*N* = 4) examined prior to and after (20 min) a furosemide injection show a significant reduction in the medullary urea signal after 34 sec (mean ± SEM).

The ^13^C MRI images of hyperpolarized urea showed time‐dependent distribution patterns. An initial perfusion of the cortex (10–20 sec) followed by large accumulation of urea localized to the medullary and pelvic regions (32–36 sec), this distribution was similar to the sodium distribution (Fig. 2). A statistically significant intrarenal corticomedullary increase in urea distribution (*P* < 0.0001) was found in both the diabetic group and the control group concomitantly with a similar corticomedullary increase in the sodium distribution in both groups (*P* < 0.0001). In both cases, the intrarenal urea and sodium gradient were found to be similar between the healthy and early diabetic kidneys (urea: *P* = 0.96 and sodium: *P* = 0.62), respectively (Fig. 3). Likewise, the cortical blood perfusion was found to be similar between the control and the diabetic kidney (*P* = 0.98), represented by the (AUC cortex/AUC aorta) signal (Fig. 4). A statistically significant reduction in the medullary urea accumulation (*P* = 0.025) following furosemide treatment was found (Fig. 5), whereas no significant perfusion change was observed (*P* = 0.42).

## Discussion

This study demonstrates that the sodium and urea gradients show a significant difference in gradient steepness as seen in Figure 3 (normalized to the cortical concentration), as described previously (Knepper et al. 2008). The main finding of this study is that hyperpolarized MR can be used to monitor the corticomedullary sodium and urea concentration gradients in diabetic and healthy rats, and more importantly that the diabetic kidney function is unaltered in the hypertrophic diabetic kidney in the early phase of diabetes. This indicates that the deranged oxygen metabolism following the prolonged hyperglycemia (Laustsen et al. 2013, 2014) does not alter the intrarenal electrolyte distribution 8–10 days after the induction of diabetes, nor do we see any effects on the mRNA transcription of kidney injury markers like KIM‐1 and NGAL, or proinflammatory markers like TNF‐*α* and IL‐1. Likewise, profibrotic or fibrotic markers like *α*‐SMA and collagen‐1 showed no difference. No oxidative stress defense on the transcriptional level has been mounted because of the unaltered Nqo‐1 level. This further supports that in this early onset of diabetes, very few alterations are seen, besides the earliest clinical signs of diabetic nephropathy including hypertrophy and albuminuria.

The sustained intrarenal electrolyte distribution, while the electrolyte transport efficiency is lowered, leads to increased renal oxygen consumption, eventually causing general hypoxia and end‐stage kidney failure (Palm and Nordquist 2011). Previous studies have demonstrated that the cortical blood perfusion is the same in healthy rats as in the STZ type‐1 diabetic rats (Palm et al. 2005). As previously shown by von Morze et al. (2012), hyperpolarized urea is appealing for renal investigations where renal changes were observed during changes in diuresis. Similarly, an intrarenal effect was found in the present study, showing the acute observable change in the medullary urea accumulation 20 min following furosemide treatment. Persistent albuminuria is currently the earliest sign of diabetic nephropathy in type‐1 diabetes (American Diabetes Association, 2003), and even though mircroalbuminuria is a highly sensitive marker, an imaging method would increase the specificity toward the high‐risk patients and enable the clinicians to take actions to protect the kidneys even before mircroalbuminuria and proteinuria. Although we did not measure the albumin content in this study, it is believed that these rats do exhibit albuminuria and thus already show sign of renal damage and as previously reported show a deranged metabolic phenotype in the diabetic kidney (Laustsen et al. 2013, 2014), thus we believe this study highlights the potential of combining ^23^Na, hyperpolarized ^13^C urea and metabolic ^13^C pyruvate MR with standard ^1^H anatomical and functional MR providing a more complete overview of functional parameters that may influence normal kidney function and contribute to development of chronic kidney disease.

## Conflict of Interest

None declared.
