# Erratum

**DOI:** 10.14814/phy2.12797

**Published:** 2016-05-04

In (Qi et al., 2016), the captions of Figures 3 and 4 were switched. Below are the correct captions:

Figure 3: The intrarenal sodium and urea signals (normalized to the total renal signal) show no difference between the diabetic group and the control group, whereas a significant difference is present within each group (mean ± SEM).

Figure 4: The renal perfusion (renal compartment/aorta signal) shows no significant alterations in the early diabetic kidney compared to control kidneys (mean ± SEM).

We apologize for the errors.
